# Clinical Associations with Hospital Escalation Among COVID-19 Patients Receiving Remdesivir in a Hospital-at-Home Service: A Real-World Cohort Study

**DOI:** 10.3390/jcm14196736

**Published:** 2025-09-24

**Authors:** Manuel Mirón-Rubio, Regina de la Corte-Carmona, Amaya Palomo-Iloro, Eduardo Fernández-Carracedo, José Ramón Sevilla-Resúa, Santiago Somovilla-Moreno, Isabel Ortega-Fernández, Francisco Bas-Sanchís, María del Carmen Montero-Hernández, Irene Gutiérrez-Gómez, Rocío Estepa-Sánchez, Eduardo Oliveros-Acebes

**Affiliations:** 1 Hospital-at-Home Service, Hospital Universitario de Torrejón, Torrejón de Ardoz, 28850 Madrid, Spain; aipalomo@torrejonsalud.com (A.P.-I.); efernandez@torrejonsalud.com (E.F.-C.); ssomovilla@torrejonsalud.com (S.S.-M.); iortega@torrejonsalud.com (I.O.-F.); fabas@torrejonsalud.com (F.B.-S.); iggomez@torrejonsalud.com (I.G.-G.); restepa@torrejonsalud.com (R.E.-S.); 2 Facultad de Medicina, Universidad Francisco de Vitoria, Pozuelo de Alarcón, 28223 Madrid, Spain; 3 Internal Medicine Service, Hospital Universitario Severo Ochoa, Leganés, 28914 Madrid, Spain; reginadelacorte@gmail.com; 4 Internal Medicine Service, Hospital Universitario de Torrejón, Torrejón de Ardoz, 28850 Madrid, Spain; jrsevilla@torrejonsalud.com (J.R.S.-R.); cmontero@torrejonsalud.com (M.d.C.M.-H.); eoliveros@torrejonsalud.com (E.O.-A.)

**Keywords:** hospital-at-home, remdesivir, COVID-19, escalation of care, immunosuppression, persistent COVID-19, lactate dehydrogenase, readmission

## Abstract

**Background/Objectives**: Hospital-at-home (HaH) programs expanded hospital capacity during the COVID-19 pandemic, but data on which HaH patients receiving intravenous (IV) remdesivir may require hospital escalation is limited. We therefore aimed to explore clinical characteristics associated with escalation to inpatient care. **Methods**: Single-center, retrospective cohort of adults with confirmed COVID-19 who received IV remdesivir via a HaH service was employed (September 2020–September 2024). Primary outcome was hospital escalation. Associations between baseline variables and escalation were assessed with bivariate statistics. **Results**: Seventy-eight HaH episodes were analyzed. Hospital escalation occurred in 4 cases (5.1%); 30-day readmission after HaH discharge occurred in 4 additional cases (5.1%). Immunosuppression and persistent COVID-19 were significantly associated with escalation (*p* = 0.03 and *p* < 0.001, respectively). Patients who escalated underwent more frequent blood testing and had longer HaH stays. Classical comorbidities (age, hypertension, diabetes, obesity, pulmonary disease) were not associated with escalation. No remdesivir discontinuations due to adverse events were recorded. **Conclusions**: In this real-world HaH cohort, IV remdesivir was well tolerated with low escalation and readmission rates. Immunosuppression and persistent COVID-19 showed significant associations with escalation, suggesting the need for refined selection and closer monitoring in these subgroups. Findings are exploratory and hypothesis-generating given the small number of events.

## 1. Introduction

Since the World Health Organization declared coronavirus disease 2019 (COVID-19) a global pandemic, health systems worldwide have faced unprecedented disruption with enduring consequences [1]. Despite global stabilization, hospitals continue to experience substantial strain [2].

During the acute phases of the pandemic, two primary challenges emerged: first, the clinical severity of the disease, which posed a significant risk of death; and second, the overwhelming number of patients affected in a short period of time, resulting in hospital overcrowding and system collapse. While the first challenge led to substantial investment in research—culminating in the rapid development of vaccines and antiviral therapies—the second demanded immediate operational responses to prevent healthcare system failure [3].

To address this second challenge, healthcare systems swiftly developed and implemented strategies to manage patients outside traditional hospital settings [4]. Among these, hospital-at-home (HaH) programs played a pivotal role in relieving pressure on inpatient services while ensuring the continuity of care for patients with COVID-19 [5,6].

Despite the widespread adoption of HaH programs during the pandemic, scientific evidence guiding the management of patients with COVID-19 in this setting remains limited. Most recommendations for HaH care are grounded in expert opinion rather than supported by robust clinical data [7,8]. This gap is particularly pronounced when determining which patients may benefit from specific therapeutic interventions, such as antiviral treatment, and under what conditions these can be administered safely at home.

In this context, it is essential to identify antiviral agents that are not only clinically effective but also operationally feasible in the HaH environment. Among available options, remdesivir has emerged as a particularly suitable option. Its indication for patients with COVID-19 is supported by evidence of antiviral efficacy [9]. In addition, its favorable safety profile, limited drug interactions, and relatively manageable requirements for storage and intravenous administration make it well suited for use in HaH programs [10]. However, determining which patients are appropriate candidates for remdesivir treatment at home remains an unresolved clinical question.

Ensuring both patient safety and the long-term viability of HaH programs requires evaluating the real-world outcomes of such interventions. One of the most relevant indicators of clinical appropriateness in this context is the need for escalation of care (that is, the transfer of a patient from the HaH setting back to hospital-based care during the episode). This outcome reflects a mismatch between initial clinical assessment and the patient’s actual trajectory, underscoring the need for more precise selection criteria [11]. Identifying potential factors associated with escalation of care is therefore essential to improving clinical decision-making and enhancing patient safety in this setting [12].

As HaH programs evolve from an emergency response into a sustained model of care, understanding which patients can be safely and effectively treated in this setting remains clinically and operationally relevant, particularly for conditions like COVID-19 that may re-emerge or serve as paradigms for future acute infectious diseases.

To address this need, we conducted a study to explore variables associated with escalation of care in patients with COVID-19 treated with remdesivir in a HaH setting.

## 2. Materials and Methods

This was an observational, analytical, and retrospective study based on data from electronic health records and hospital registries of patients diagnosed with COVID-19 who received intravenous remdesivir (Gilead Sciences, Foster City, CA, USA) treatment in the HaH unit of Hospital Universitario de Torrejón (Madrid, Spain). All eligible cases treated between September 2020 and September 2024 were included using a convenience sampling approach. No formal sample size calculation was performed, as this was a retrospective, exploratory study. All eligible consecutive patients treated during the study period were included. The study protocol was reviewed and approved by the Research Ethics Committee with Medicines (CEIm) of Hospital Universitario Elche-Vinalopó (approval code CEImHUV 2025.009) on 23 April 2025.

The study was conducted within the hospital-managed HaH model implemented at Hospital Universitario de Torrejón, which provides hospital-level acute care in the home as an alternative to conventional inpatient hospitalization. The HaH unit offered continuous medical and nursing care, including parenteral treatments, complex wound care, acute palliative care, and home-based respiratory therapies. Physicians were available on-site from 08:00 to 15:30 on weekdays and on-call until 21:00 every day, including weekends. Nursing staff were present from 08:00 to 21:00 daily, with night-time coverage provided by the hospital’s on-call nursing supervisor. The program served approximately 150,000 residents within a 30 km or 35 min radius and could accommodate up to 64 simultaneous patients, with additional staffing deployed during peak demand.

Eligible patients were aged 18 years or older, had confirmed severe acute respiratory syndrome coronavirus 2 (SARS-CoV-2) infection (by antigen test or polymerase chain reaction-PCR), and received at least one dose of intravenous remdesivir during a HaH episode. Remdesivir was prescribed to immunocompetent patients with respiratory failure requiring supplemental oxygen, and to immunocompromised patients with confirmed COVID-19 regardless of oxygen requirements. All patients met the unit’s standard admission criteria, including clinical and hemodynamic stability, residence within the coverage area, 24 h caregiver availability, and telephone accessibility. Exclusion criteria included oxygen flow ≥4 L/min, known allergy to remdesivir, lack of adherence or cooperation, psychomotor agitation, or use of non-standardized experimental treatments.

The primary outcome was hospital escalation, defined as the unplanned transfer of a patient from the HaH program to inpatient care prior to the completion of the home hospitalization episode. The secondary objective was to evaluate the safety and tolerability of remdesivir administration in the HaH setting, defined as the occurrence of adverse events requiring treatment discontinuation. This outcome served as an indicator of the feasibility of antiviral therapy in the outpatient context.

The independent variables included demographic data (age, sex, and country of birth—Spain vs. non-Spain); clinical characteristics such as immunosuppression, persistent COVID-19, hypertension, diabetes, obesity, chronic pulmonary disease, and requirement for supplemental oxygen at HaH admission; and analytical parameters including C-reactive protein (CRP), ferritin, D-dimer, lymphocytes, neutrophils, platelets, lactate dehydrogenase (LDH), aspartate transaminase (AST), and alanine transaminase (ALT). Laboratory values obtained at diagnosis or within the first 48 h were included. Immunosuppression was defined as the presence of conditions or treatments associated with impaired immune function that increased susceptibility to severe or prolonged infection. Persistent COVID-19 was defined as ongoing SARS-CoV-2 infection with positive RT-PCR tests and/or persistent clinical symptoms beyond 30 days from the initial diagnosis. Additional care-related variables comprised the number of remdesivir doses administered, the number of blood tests performed during follow-up, unscheduled nursing visits, admission scheme (early discharge vs. admission avoidance), and department of referral (hospital ward, emergency department, outpatient clinic, or day hospital).

Statistical analyses were conducted to explore factors associated with hospital escalation. Continuous variables were expressed as medians and interquartile ranges (IQR) and compared using the Mann–Whitney U test. Categorical variables were summarized as counts and percentages and compared using the chi-squared test or Fisher’s exact test, as appropriate.

A *p*-value < 0.05 was considered statistically significant. Data were analyzed using Python (Python Software Foundation, Wilmington, DE, USA; version 3.11.8) with the pandas (version 1.5.3), statsmodels (version 0.13.5), and scipy (version 1.14.1) libraries, and R (R Foundation for Statistical Computing, Vienna, Austria; version 4.4). Results were reported in accordance with the STROBE (Strengthening the Reporting of Observational Studies in Epidemiology) guidelines.

During the preparation of this manuscript, the authors used a generative artificial intelligence tool, ChatGPT (OpenAI, GPT-4), to assist with the interpretation of statistical results and to improve the clarity and readability of the text. All AI-generated content was critically reviewed, edited, and verified by the authors, who take full responsibility for the accuracy and integrity of the final manuscript.

## 3. Results

A total of 78 patient episodes involving 77 individuals were included in the analysis. Among these, 4 patients (5.1%) required escalation to inpatient hospital care during the HaH episode, and an additional 4 patients (5.1%) were readmitted within 30 days after discharge.

### 3.1. Patient Characteristics and Overall Outcomes

Baseline characteristics are presented in Table 1. The cohort had a mean age of 69.8 years (range: 39–95, median: 72, IQR: 60–80), and 61% were male. Common comorbidities included hypertension (46.7%), pulmonary disease (45.4%), diabetes mellitus (24.6%), obesity (15.5%), and immunosuppression (20.7%). A total of 6.4% were diagnosed with persistent COVID-19. Most patients (74.4%) were admitted to HaH via early discharge from inpatient care, and 30.7% required supplemental oxygen at HaH admission. The mean duration of HaH episodes was 8 days, with a median of 6 days (IQR: 5–8.8), and patients received an average of 2.8 doses of remdesivir (median; 2.5, IQR: 2–4).

Overall, 93.6% of episodes resulted in a favorable clinical outcome, with only 1 death recorded (1.3%). Emergency room visits without escalation occurred in 2 cases (2.6%).

### 3.2. Clinical and Analytical Differences in Escalated Cases

Patients who required hospital escalation showed a significantly longer median duration of HaH stay (13 vs. 6 days, *p* = 0.049) and underwent more frequent blood testing (median 2 vs. 0 tests; *p* = 0.01) (Table 2).

The presence of immunosuppression was also significantly associated with escalation (*p* = 0.03). Detailed in Table 3, the most frequent causes of immunosuppression were hematologic malignancies, accounting for 68.8% of immunosuppressed patients, including chronic lymphocytic leukemia (CLL), myelodysplastic syndrome, and multiple myeloma. Both patients with CLL had advanced disease and were considered immunocompromised based on clinical progression and hematologic risk factors, including TP53 mutations and history of infectious complications. Other cases included human immunodeficiency virus (HIV) infection, primary immunodeficiencies, and patients receiving immunosuppressive therapy due to autoimmune diseases (e.g., systemic lupus erythematosus, Behçet’s disease) or solid organ transplantation.

Persistent COVID-19 was strongly associated with escalation (*p* < 0.001). All patients with persistent COVID-19 in this cohort also had an underlying immunosuppressive condition.

LDH levels were higher among patients who escalated (median 568.5 vs. 347 U/L), showing a tendency towards a significant difference compared to non-escalated patients *p* = 0.06). Other biomarkers such as CRP, ferritin, and lymphocyte or neutrophil counts did not show significant associations with escalation status.

### 3.3. Lack of Association with Traditionally Reported Risk Factors

Variables traditionally described as risk factors in COVID-19—such as age, hypertension, diabetes, obesity, and pulmonary disease—did not show significant associations with escalation in our cohort (all *p* > 0.05), likely reflecting the effects of stringent HaH selection criteria (Table 2).

## 4. Discussion

In this real-world cohort of COVID-19 patients treated with intravenous remdesivir at home, we observed low rates of hospital escalation and post-discharge readmission. Escalation was significantly associated with immunosuppression and persistent COVID-19, whereas classical comorbidities such as age, hypertension, diabetes, and obesity were not. Patients who required escalation had longer HaH stays, underwent more frequent laboratory testing, and showed a trend toward higher LDH values. No treatment discontinuations due to adverse events were observed, underscoring the safety of remdesivir in this setting.

The rate of hospital escalation during the active episode of HaH was 5.1%. Our findings are consistent with those reported in other HaH studies for patients with COVID-19. Pereta et al. described escalation rates of 5% in patients treated with intravenous remdesivir [13], while outpatient studies without continuous supervision reported low escalation rates of 1.3–2.8% [14,15,16]. Similarly, in HaH models not specifically based on remdesivir therapy, Llorens et al. reported a 6.4% escalation rate [17], Pericàs et al. 4.8% [18], and Sánchez-Fabra et al. 10.2% [19]. These results suggest that when patients are carefully selected and monitored, HaH programs can safely manage moderate COVID-19 cases, with escalation rates comparable across different settings and models.

Additionally, our observed 5.1% readmission rate is also comparable to reported 30-day readmission rates after inpatient COVID-19 care, which range from 3% to over 10% depending on population and setting [20,21,22,23,24,25,26,27].

Importantly, previous outpatient cohorts of remdesivir therapy also showed very low rates of adverse events and treatment discontinuation [14,16,28,29], consistent with our finding that no discontinuations occurred in this series.

One patient died during HaH, a frail resident of a long-term care facility whose family declined hospital transfer. This case illustrates the need for individualized decision-making in end-of-life contexts, where HaH may also serve as a palliative option. Notably, Sánchez-Fabra et al. reported a mortality rate of 18.6% in an elderly and institutionalized HaH cohort [19], emphasizing that mortality in HaH often reflects frailty rather than failure of the model.

A particularly relevant finding was the association between immunosuppression and escalation. This aligns with prior evidence showing increased severity, prolonged viral shedding, and higher mortality among immunosuppressed COVID-19 patients [30]. In our cohort, hematologic malignancies accounted for most cases, but other conditions such as HIV, primary immunodeficiencies, or chronic immunosuppressive therapy were also represented. These patients are especially vulnerable to deterioration even under close monitoring, consistent with previous HaH studies in transplant recipients [31] and reports of HaH failure in patients with profound B-cell depletion despite antiviral therapy [32].

Persistent COVID-19 was also significantly associated with escalation, but in all cases occurred in immunocompromised patients, suggesting that it is not an independent predictor but rather a clinical expression of impaired immunity [32]. This overlap highlights the intertwined risk of immunosuppression and persistent infection as contexts for escalation.

Although LDH did not reach statistical significance, patients who escalated showed higher baseline values, consistent with its role as a marker of tissue injury and worse trajectories in hospitalized patients [30,33]. In our cohort, LDH was measured during the initial hospital stay and not at the time of HaH admission. This timing limits its immediate utility as a triage tool for HaH eligibility. However, it underscores its potential value as a retrospective indicator of disease severity that may not be captured by clinical presentation or vital signs alone [34]. Beyond LDH, other inflammatory markers have also been associated with outcomes in HaH. For example, Bonet-Papell et al. found that higher levels of CRP and high-sensitivity troponin T (Hs-TnT) at the start of HaH were associated with increased risk of readmission [35].

Escalated patients also underwent repeated blood testing and had longer HaH stays. These factors likely reflect evolving clinical deterioration rather than baseline predictors, but prolonged stays may also indicate delayed decisions to escalate, underscoring the need for structured reassessment and timely transfer in HaH.

Contrary to findings in many hospitalized COVID-19 cohorts, no statistically significant association was observed between classical comorbidities—such as advanced age, hypertension, diabetes mellitus, or obesity—and hospital escalation, which likely reflects the effects of rigorous patient selection [25,26,27,36]. Indeed, HaH programs typically rely on inclusion criteria that preclude patients with unstable chronic disease, frailty, or uncontrolled comorbidities. As a result, the range of risk conveyed by these baseline variables may have been attenuated in our sample. In contrast to our study, Chou et al. identified overall comorbidity burden as a predictor of escalation risk, although specific conditions like age and obesity were not independently associated, possibly reflecting similar selection biases in HaH eligibility [37].

To our knowledge, this represents one of the largest reported series specifically evaluating remdesivir administration in a HaH setting. The findings of this study highlight several key variables that may inform risk stratification and operational decision-making in COVID-19 patients eligible for home HaH. In particular, the presence of immunosuppression—especially in patients with hematologic malignancies—should prompt critical reassessment of current inclusion criteria for HaH.

In addition, recent literature supports the relevance of integrating both clinical status and structured predictive tools in discharge and escalation protocols. Finn et al. demonstrated that normalization of vital signs within 24 h prior to discharge was significantly associated with a lower risk of 30-day readmission in COVID-19 patients [34]. Moreover, Gavin et al. validated the use of the simplified HOSPITAL score in COVID-19 patients and found it to be a pragmatic tool for predicting 30-day potentially avoidable readmissions, including among those discharged to HaH programs [38]. Importantly, patients discharged to HaH with low scores had the lowest observed readmission rates (1.9%), suggesting that such tools may help refine eligibility criteria and safely expand HaH utilization without compromising outcomes.

From an operational perspective, our findings support the implementation of early warning systems and predefined escalation protocols within HaH. Structured reassessment timelines, combined with flexible criteria for transfer to acute care, may reduce the risk of delayed escalation and associated complications. Additionally, tracking dynamic indicators such as repeat laboratory testing or persistent symptomatology can be used to trigger alerts, resource reallocation, or higher-frequency visits.

This study has limitations. The small number of escalations (n = 4) precluded robust multivariable analyses and effect size estimation. Advanced regression models were attempted but proved statistically unstable, so only descriptive and bivariate analyses were reported. Future studies with larger cohorts are needed to validate our findings and allow for robust multivariable modeling to account for potential confounders.

The retrospective, single-center design and selection bias inherent to HaH also limit generalizability. Additionally, imaging data (e.g., CT severity scores) were not consistently collected. While these are valuable predictors for COVID-19 severity, their absence in our dataset precluded their inclusion. This limitation should be considered when interpreting our findings.

Despite these limitations, the study offers valuable insights into the profile and clinical characteristics associated with escalation among patients treated with remdesivir in a HaH setting and highlights potential avenues for refining patient selection and monitoring strategies.

## 5. Conclusions

This study provides real-world evidence on the use of intravenous remdesivir in HaH, showing low hospital escalation and readmission rates. Immunosuppression and persistent COVID-19 were significantly associated with escalation, although causality cannot be inferred, while classical comorbidities showed no predictive value. These findings highlight the importance of refined patient selection and individualized monitoring to optimize outcomes in home-based COVID-19 care.

Although the burden of acute COVID-19 hospitalizations has declined, the insights from this study remain relevant. HaH programs are increasingly being integrated into long-term healthcare delivery beyond the pandemic, making clear selection, monitoring, and escalation criteria essential. Lessons learned from this pandemic may also apply to other respiratory viral infections and future scenarios requiring decentralized models of acute care. Further research with adequately powered samples is required to confirm these associations and build predictive models for safe patient selection in HaH settings.

## Figures and Tables

**Table 1:** Baseline characteristics and clinical outcomes of COVID-19 patients treated with remdesivir in hospital-at-home (*n* = 77 patients; 78 HaH episodes).

Characteristic	Value
Sex, *n* (%)	
Male	47 (61)
Female	30 (39)
Age (years), median (IQR)	72 (60–80)
Male	73 (62.5–80)
Female	69.5 (55.5–80)
Nationality, *n* (%)	
Spanish	63 (81.8)
Non-Spanish	14 (18.2)
Comorbidities, *n* (%)	
Diabetes	19 (24.6)
Hypertension	36 (46.7)
Immunosuppression	16 (20.7)
Obesity	12 (15.5)
Pulmonary disease	35 (45.4)
Persistent COVID-19, *n* (%)	5 (6.4)
Supplemental oxygen required at HaH admission, *n* (%)	24 (30.7)
Requesting service, *n* (%)	
Internal Medicine	61 (78.2)
Emergency Department	12 (15.4)
Pulmonology	3 (3.8)
Hematology	2 (2.6)
Admission scheme, *n* (%)	
Early discharge (Internal Medicine)	58 (74.4)
Admission avoidance	20 (25.6)
Emergency Department	12 (15.3)
Outpatient Clinic	6 (7.7)
Day Hospital	2 (2.6)
Remdesivir treatment	
Total doses administered, *n*	221
Median (IQR)	2.5 (2–4)
Other IV treatments, *n* (%)	22 (28.2)
HaH length of stay (days)	
Total HaH stays	627
Median (IQR)	6 (5–8.8)
HaH outcomes, *n* (%)	
Favorable clinical outcome	73 (93.6)
Inpatient care escalation	4 (5.1)
ER visits (non-escalation)	2 (2.6)
Deaths	1 (1.3)
Adverse events requiring remdesivir discontinuation	0 (0)
30-day readmission after discharge, *n* (%)	4 (5.1)

Data are presented as median (IQR) or n (%). IQR: interquartile range; HaH: hospital-at-home; IV: intravenous; ER: emergency room.

**Table 2:** Clinical and analytical characteristics by escalation status in COVID-19 patients treated with remdesivir in hospital-at-home.

Variable	Escalation (*n* = 4)	No Escalation (*n* = 74)	*p*-Value (Bivariate)
Male sex, *n* (%)	2 (50)	45 (61.6)	1.00
Age (years), median (IQR)	74.5 (59.5–89.5)	72 (60–80)	0.58
Nationality: Spanish, *n* (%)	3 (75)	60 (81.1)	1.00
Comorbidities, *n* (%)			
Diabetes	0 (0)	19 (26)	0.56
Hypertension	3 (75)	33 (45.2)	0.52
Immunosuppression	3 (75)	13 (17.6)	0.03
Obesity	1 (25)	11 (15.1)	1.00
Pulmonary disease	1 (25)	34 (46.6)	0.74
Persistent COVID-19, *n* (%)	3 (75)	2 (2.7)	<0.001
Supplemental oxygen required, *n* (%)	3 (75)	21 (28.4)	0.16
Department of referral, *n* (%)			
Hospital ward	3 (75)	55 (74.3)	0.49
Emergency department	0 (0)	12 (16.2)	
Outpatient clinic	1 (25)	5 (6.8)	
Day hospital	0 (0)	2 (2.7)	
Admission scheme, *n* (%)			1.00
Early discharge	3 (75)	55 (74.3)	
Admission avoidance	1 (25)	19 (25.7)	
Biomarkers, median (IQR)			
AST [U/L]	27 (20.2–46)	27 (21–37)	0.96
ALT [U/L]	37.5 (26.2–54.8)	29 (21–43)	0.41
C-reactive protein (mg/L)	37.1 (12.9–59.9)	42.6 (22.5–89.9)	0.42
D-dimer (ng/mL)	682 (391.5–2429)	545.5 (424–811.5)	0.87
Ferritin (ng/mL)	345.5 (167.5–694.5)	269 (161–485.2)	0.92
LDH (U/L)	568.5 (401.2–929.2)	347 (236–512)	0.06
Lymphocytes (×10^9^/L)	0.9 (0.7–24)	1.2 (0.7–1.7)	0.64
Neutrophils (×10^9^/L)	6.4 (5.2–7.6)	5.1 (3.5–6.7)	0.32
Platelets (×10^9^/L)	175.5 (129.5–213.5)	181 (143–233)	0.51
Blood tests performed, median (IQR)	2 (1.8–3)	0 (0–1)	0.01
Blood tests ≥ 2, *n* (%)	3 (75)	8 (11)	0.01
No. of remdesivir doses, median (IQR)	4 (3.5–4.2)	2 (2–3)	0.12
Other IV treatment, *n* (%)	1 (25)	21 (28.4)	1.00
HaH stay duration, (days), median (IQR)	13 (9–17.8)	6 (5–8)	0.049
Unscheduled nursing visits, median (IQR)	0 (0–0.2)	0 (0–0)	0.20

Bivariate *p*-values were calculated using Mann–Whitney U test or chi-squared/Fisher’s exact test, depending on variable type. *p* < 0.05 was considered statistically significant. Data are presented as median (IQR) or *n* (%). HaH: hospital-at-home; LDH: lactate dehydrogenase; CRP: C-reactive protein; AST: aspartate transaminase; ALT: alanine transaminase.

**Table 3:** Causes and diagnoses of immunosuppression among COVID-19 patients in hospital-at-home (*n* = 78 episodes).

Condition	*n* (%)
Hematologic	11 (14.1)
Chronic lymphocytic leukemia ^†^	3 (3.8)
Myelodysplastic syndrome	2 (2.6)
Common variable immunodeficiency	1 (1.3)
Acute myeloid leukemia	1 (1.3)
Follicular lymphoma	2 (2.6)
Marginal zone lymphoma	1 (1.3)
Multiple myeloma	1 (1.3)
Immunosuppressive therapy	4 (5.1)
Multiple sclerosis	1 (1.3)
Behçet’s disease	1 (1.3)
Systemic lupus erythematosus	1 (1.3)
Kidney transplant	1 (1.3)
HIV infection	1 (1.3)

Note: ^†^ Two of the three HaH episodes involving patients with CLL correspond to the same individual. Both patients had advanced diseases and were considered immunocompromised due to marrow involvement and recent immunochemotherapy. HaH: hospital-at-home; CLL: chronic lymphocytic leukemia.

## Data Availability

The data supporting the findings of this study are not publicly available due to privacy and ethical restrictions. Anonymized data may be available from the corresponding author upon reasonable request and with approval from the Research Ethics Committee.

## Abbreviations

The following abbreviations are used in this manuscript:

HaH	Hospital-at-Home
COVID-19	Coronavirus Disease 2019
LDH	Lactate Dehydrogenase
AST	Aspartate Transaminase
ALT	Alanine Transaminase
CRP	C-Reactive Protein
PCR	Polymerase Chain Reaction
IQR	Interquartile Range
CEIm	Comité de Ética de la Investigación con medicamentos
HIV	Human Immunodeficiency Virus
IV	Intravenous
ER	Emergency Room
